# Love Addiction, Adult Attachment Patterns and Self-Esteem: Testing for Mediation Using Path Analysis

**DOI:** 10.3390/jpm13020247

**Published:** 2023-01-29

**Authors:** Alessio Gori, Sara Russo, Eleonora Topino

**Affiliations:** 1 Department of Health Sciences, University of Florence, Via di San Salvi 12, Pad. 26, 50135 Florence, Italy; 2 Integrated Psychodynamic Psychotherapy Institute (IPPI), Via Ricasoli 32, 50122 Florence, Italy; 3 Department of Human Sciences, Libera Università Maria Santissima Assunta (LUMSA), Via della Traspontina 21, 00193 Rome, Italy

**Keywords:** love addiction, anxious attachment, adult attachment, self-esteem, self-concept, path analysis, mediation

## Abstract

Love addiction is a dysfunctional relational modality that takes on the addiction characteristics and which, for the individuals who suffer from it, can have a negative and pervasive impact on various areas of functioning. The objective of this research was the analysis the factors that can be associated with love addiction, particularly focusing on adult attachment patterns and self-esteem. A sample group of 300 individuals who declared themselves to have a romantic relationship was involved in this research (*M_age_* = 37.83 years, *SD* = 12.937). They completed an online survey including the Love Addiction Inventory—Short form, Relationship Questionnaire, and Rosenberg Self-Esteem Scale. Results showed significant and positive associations between preoccupied and fearful adult attachment and love addiction. Furthermore, these relationships were totally mediated by self-esteem. Gender and age were controlled as potential covariates and showed significant effects in influencing the levels of self-esteem and love addiction. Such findings may provide useful information for orienting future research and supporting an effective clinical practice.

## 1. Introduction

Love addiction is one of the “New Addictions”, a heterogeneous set of disorders that shares psychopathological characteristics with substance dependence, characterized by a spasmodic research of the object of dependence without which existence seems meaningless [1]. Despite the attempts at definition and the etiological hypotheses, this condition is not included in any official nosographic system, e.g., *DSM-5* [2]. Love addiction can be defined as a maladaptive and problematic model of the love relationship, characterized by a pervasive and excessive interest in the partner, with consequent loss of control and continuation of the relationship despite the awareness of the existence of problems created by the relationship itself [3,4]. This condition is featured by an alteration of the representation of self, as an extremely needy subject of guidance, support, and protection, and of the other (the object of dependence) being extremely idealized [5]. Reynaud [4] has identified the crucial moment of transition from healthy love to pathological love when desire becomes need, suffering takes precedence over pleasure, lack becomes paramount, and when the relationship continues despite the negative consequences. The love addicted subject often feels inadequate, unworthy of love, and lives in constant fear of being abandoned by his partner. It is precisely the fear of abandonment that leads to the exacerbation of attempts to control others, with complacent behavior, sacrifice, availability, and care, in the hope that the relationship will become stable and lasting [6]. A model that has identified the fundamental components that constitute love addiction is the ‘components’ model of addiction by Griffiths [7]. The six constructs of the model are as follows: *salience*, i.e., directing almost all feelings, thoughts and behaviors towards the object of love; *tolerance*, i.e., an increasing need for time spent with, and/or time spent thinking about the loved one; *mood modification*, i.e., coping with emotional distress by spending time with and/or thinking about the object of love; *relapse*, i.e., difficulty reducing the time spent in the presence of a loved one; *withdrawal*, i.e., typical both psychological and physical withdrawal symptoms in the absence of the loved one; and *conflict*, i.e., interference with daily activities, such as work and education, friendships, activities and hobbies. There is evidence in the scientific literature on the negative consequences of this behavioral dependence, which is why a more in-depth study of this issue is needed. Love addiction is accompanied by a reduction in social, professional, and leisure activities [4], resulting in a lower quality of life; moreover, when associated with stressful life events, it can lead to a high risk of medical and psychological pathologies [8]. Subjects with love addiction may have high levels of depression and alexithymia [9] and a possible comorbidity with post-traumatic stress disorder [10] and with other types of addiction, such as sex addiction [4]. Moreover, interpersonal dependence would seem to be a factor that plays a role of strong influence on body perception, resulting in dissatisfaction with the body and unregulated nutrition [11].

### 1.1. Love Addiction and Attachment 

Considering the strong impact and negative consequences of love addiction, the study of related factors seems particularly relevant. A first factor that can play a fundamental role in addiction is attachment. Attachment can be defined as an emotional bond in which an individual seeks the closeness of the attachment object and uses it as a safe place during times of distress and as a safe base from which to explore the world [12]. John Bowlby, in his theory of attachment [13], conceptualized the human tendency to establish emotional ties with significant others. According to Bowlby’s theory, individuals in childhood internalize experiences with their caregivers, establishing an early attachment, which lays the foundations for future relational modalities, outside the family [14]. As such, the attachment style becomes reactivated in close relationships during late adolescence and early adulthood [15]. There are individual differences in the quality of those attachments. Bartholomew and Horowitz [16] described the different attachment styles (secure, insecure preoccupied, insecure dismissing, and insecure fearful) in their “Model of Adult Attachment”, derived from a combination of two dimensions, namely “Model of self” and “Model of other”. A secure attachment style is characterized by a sense of lovability and expectation that other people will be available and responsive. Individuals with such attachment style are comfortable with intimacy and autonomy, are more optimistic about life, use more effective emotion regulation strategies, can remain open to their emotions and can express them freely to others [16,17].

A preoccupied attachment style is instead characterized by a sense of inadequacy, combined with a positive assessment of others. Individuals with such attachment style attempt by obtaining the acceptance of others to increase acceptance for themselves, indeed they search anxiously for love and support [16,18]. The third style of attachment, dismissing, implies a sense of inadequacy and the expectation that others are unavailable, and unreliable. Not feeling worthy of love, individuals with such attachment style avoid close involvement, to protect themselves from the rejection of others. The fourth and last attachment style, the fearful one, indicates a sense of lovability combined with a negative disposition towards others. Individuals with such attachment style avoid intimate relationships while maintaining a sense of independence and invulnerability, to protect themselves from disappointment. While both dismissing and fearful styles reflect avoidance of intimacy, they differ in the need for acceptance by others to maintain positive self-esteem; such a need is present in the dismissing style, but not in the fearful one. Similarly, the preoccupied and fearful styles share strong dependence on others but differ in willingness to establish intimate relationships, as preoccupied individuals actively seek closeness with others, while fearful individuals tend to avoid it [16].

Evidence in the literature shows that a type of insecure attachment is a strong risk factor for substance use disorders [19] but also for symptoms of behavioral addictions [20], such as gambling disorder [21], problematic shopping [22], problematic social media use [23], and sexual addiction [24]. Insecure attachment is also associated with love addiction in the literature and, therefore, to a vision of love characterized by obsession and dependence, confirming the hypothesis that the dynamics of attachment are not limited to childhood, but rather remain throughout the course of life [25,26,27]. 

### 1.2. Love Addiction and Self-Esteem

Another factor of great importance, which can be analyzed in relation to love addiction, is self-esteem. The latter is defined in the literature as a fundamental component of the concept of self, since it constitutes its affective orientation, understood as a positive or negative assessment of itself. This construct, therefore, appears to be in close connection with the feeling of personal value, and would seem to play a determining role in the development of coping skills and a sense of well-being [28,29]. In the relationship between self-esteem and attachment, studies that have analyzed this association have shown that a secure attachment style is related to higher levels of self-esteem than an insecure attachment style. In particular, secure attachment is associated with the “Broaden and build circle” technique, which would contribute to a positive association between self-esteem and clarity of the concept of self. Conversely, an insecure style of attachment would seem to be associated with lower levels of self-esteem and clarity of the concept of self, as people classified as insecure are less effective in using and providing a safe and consistent basis for their partners, have less satisfaction and more conflict in relationships and also report lower self-esteem [30,31,32]. Referring to the “Model of adult attachment” [16], data in the literature highlight how secure and dismissing attachment styles are associated with higher global self-esteem and greater average competence in the social, romantic, physical attractiveness, and sport domains, compared to either preoccupied or fearful attachment styles [33].

In the literature, it also emerges how self-esteem has a role in behavioral addictions. Indeed, studies show that subjects with Internet and Facebook addiction have low levels of self-esteem and show how the latter plays the role of predictor of an Internet addiction [34,35]. Low self-esteem is also a contributing factor to the formation of interpersonal dependence and depressive symptoms [36]. Furthermore, insecure attachment and low self-esteem may heighten feelings of insecurity about the relationship and could manifest as obsessive love; studies show that subjects involved in pathological love have lower levels of self-esteem and self-directedness than subjects with healthy relationships [31,37]. 

### 1.3. The Present Research

Given the aforementioned evidence, this research investigated the relationships between factors that may be associated with love addiction, focusing on adult attachment, and self-esteem. Therefore, the relationship between secure attachment, fearful attachment, preoccupied attachment, and dismissing attachment, and love addiction first were explored. Then, considering only the attachment styles showing a significant total effect, a path analysis was performed, hypothesizing that:

**Hypothesis 1 (H_1_).**  *Adult attachment patterns would be significantly associated with self-esteem*.

**Hypothesis 2 (H_2_).**  *Self-esteem would be significantly associated with love addiction*.

**Hypothesis 3 (H_3_).**  *Self-esteem would be a significant mediator in the relationship between adult attachment patterns and love addiction*.

Since previous studies have shown the influence of age and gender on the considered variables [4,38,39], these factors were controlled as covariates to test the solidity of the interactions hypothesized in the model.

## 2. Materials and Methods

### 2.1. Participants and Procedure

A sample group of 300 Italian individuals who declared themselves to have a romantic relationship was involved in this research (see Table 1). 

Of the sample group, 42% were married, while 58% were only cohabiting. Their mean age was 37.83 years (*SD* = 12.937). Most of them were women (80%) and had a high school diploma (38%). They were recruited on the Internet through a snowball-like sampling method. The inclusion criteria were as follows: (1) having a romantic relationship; (2) being at least 18 years old. Each participant completed the survey online, through the Google Forms platform, after being briefed about the general aim of the research and providing informed consent electronically. Privacy and anonymity were guaranteed. All the procedures of this research were approved by the first author’s institutional ethical committee.

### 2.2. Measures

#### 2.2.1. Love Addiction Inventory—Short Form (LAI—SF)

The *Love Addiction Inventory*—*Short Form* (LAI—SF) [40] is a 6-item self-report scale used for the assessment of the levels of love addiction, based on the components model of behavioural addiction [7]. Items are scored on a 5-point Likert scale, from 1 (never) to 5 (very often). Higher scores indicate higher levels of love addiction symptoms. The original Italian 6-item version [40] was used in this research and showed an acceptable internal consistency in the present sample (*α* = 0.67).

#### 2.2.2. Relationship Questionnaire (RQ)

The *Relationship Questionnaire* (RQ) [16,41] is a 4-item self-report scale used for the assessment of adult attachment patterns. Items are scored on a 7-point Likert scale, from 1 (“It does not describe me at all”) to 7 (“It very much describes me”) and allow for the assessment of secure, dismissing, preoccupied, and fearful attachment styles. For each item, higher scores indicate higher levels of the corresponding attachment style. The Italian version was used in this research [41] and, since the four attachment styles are assessed with a single item, the alpha coefficient cannot be calculated. Nevertheless, RQ was found to have good test–retest reliability [42] and good psychometric properties in different cultures [43]. 

#### 2.2.3. Rosenberg Self-Esteem Scale (RSES)

The *Rosenberg Self-Esteem Scale* (RSES) [44,45] is a 10-item self-report scale used for the assessment of the levels of global self-esteem. Items are scored on a 4-point Likert scale, from 0 (strongly agree) to 3 (strongly disagree). Higher scores indicate higher self-esteem. The total score of the Italian version was used in this research [45] and showed good internal consistency in the present sample (*α* = 0.84).

### 2.3. Statistical Analyses

Statistical analyses were performed using SPSS (v. 21.0; IBM, New York, NY, USA) and AMOS (v. 24.0; IBM, New York, NY, USA) for Windows. Pearson correlations were performed to explore the association among the variables of this research. Only the attachment patterns which were significantly associated with love addiction were included in the mediation model, which was implemented by using a path analysis [46], testing the mediation of self-esteem in the relationship between adult attachment and love addiction, controlling for the effects of gender (with males coded as “0” and females coded as “1”) and age as potential confounders. The statistical fit of the model was evaluated based on the following goodness-of-fit indicators: the discrepancy divided by degree of freedom (CMIN/DF), indicating a reasonable fit for values less than 5 [47]; the goodness of fit (GFI), indicating a reasonable fit for values above 0.90 [48]; the normed-fit index (NFI), indicating a reasonable fit for values above 0.90 [49]; and the standardized root mean square residual (SRMR), indicating a reasonable fit for values less than 0.08 [50]. The statistical stability and significance of the model was further investigated by performing the bootstrapping procedure for each of the 5000 bootstrapped samples with a 95% confidence interval [51].

## 3. Results

As shown in Table 2, love addiction was negatively and significantly correlated to self-esteem (*r* = 0.364, *p* < 0.01), and positively and significantly associated with preoccupied (*r* = 0.203, *p* < 0.01) and fearful (*r* = 0.256, *p* < 0.01) adult attachment.

**Figure 1:**
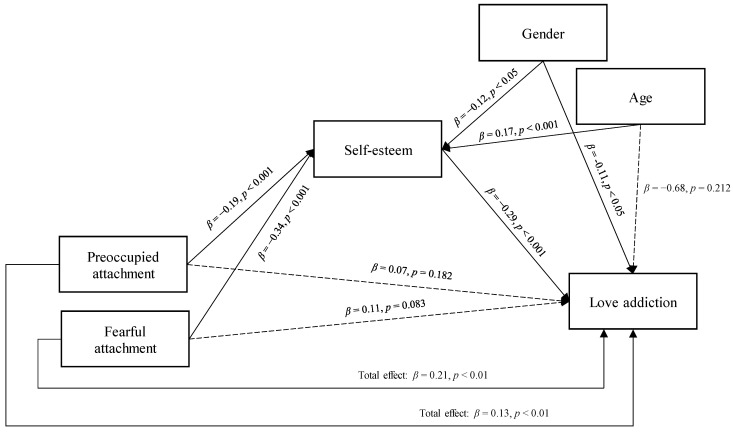
The mediation of self-esteem in the relationship between preoccupied and fearful adult attachment and love addiction, controlling for the effects of gender and age—a path analysis model. Note—dashed lines indicate the non-significant direct effects.

On that basis, a path analysis was implemented to test the mediation of self-esteem in the relationship between preoccupied and fearful adult attachment, and love addiction, controlling for the effects of gender and age (see Figure 1). The emerging mediation model showed a good fit, as follows: CMIN/DF = 4.501, GFI = 0.921, NFI = 0.907, and SRMR = 0.065.

More specifically, preoccupied and fearful attachment patterns were significantly and negatively associated with self-esteem (*β* = −0.019, *p* < 0.001 and *β* = −0.34, *p* < 0.001, respectively; **H_1_**). In turn, self-esteem was significantly and negatively related to love addiction (*β* = −0.29, *p* < 0.001; **H_2_**). With regards to the covariates, while being female was associated with low levels of self-esteem (*β* = −0.12, *p* < 0.05) and high levels of love addiction (*β* = 0.11, *p* < 0.05), being older was related to higher levels of self-esteem (*β* = 0.17, *p* < 0.001), while no significant effects have been highlighted between age and love addiction in this model (*p* = 0.212). Finally, when included in the model, self-esteem totally mediated the effect of preoccupied and fearful attachment on love addiction, determining non-significant direct effects (*β* = 0.07, *p* = 0.182 and *β* = 0.11, *p* = 0.083, respectively, **H_3_**).

The bootstrapping procedure confirmed the statistical stability of the mediation model (see Table 3).

## 4. Discussion

In recent years love addiction has become the subject of a strong interest within the scientific community. Recent studies show its similarity to other forms of addiction, such as substance dependence [52]. This data, based on behavioral, neurochemical, and neuroimaging evidence, underlines the importance of focusing on this type of addiction that is accompanied, due to its pervasive nature, by a strong negative impact on the life of those who suffer from it, including a high level of psychological suffering and loss of control. In fact, individuals with love addiction tend to experience negative mood and affective states when they are away from their partner and have the strong need and desire to see them deal with stressful situations [3,27]. Given the clinical relevance of the phenomena, the present study aimed at providing further knowledge on the psychological factors that may be associated with love addiction, by specifically focusing on adult attachment styles and self-esteem.

Concerning the relationship between love addiction and adult attachment, our results showed that only fearful and preoccupied styles showed significant and positive associations with love addiction. This is in line with earlier evidence showing how fearful and preoccupied attachments are associated with different forms of addiction; these two styles of attachment seem to be more frequent in subjects with substance dependence, such as with heroin [19], alcohol, tobacco, or waterpipes [53], as well as problematic smartphone use [54] and social media addiction [23,55], and sexual addiction [56]. The preoccupied attachment is characterized by fear that others are unavailable in times of need, and involves an anxious search for love and support and dependence on a desire for commitment in relationship, while fearful attachment is characterized by distrust of others’ intentions, compulsive self-reliance, and the low intensitiy of fewer love experiences [18,25]. Both arise from an attempt, in childhood, to adapt to a figure of attachment available in a way that is inconsistent or constantly unavailable. Such modalities become maladaptive when applied to later relationships, where the search for support and comfortable interdependence could be rewarding and help the person maintain a sense of well-being [18]. Such styles, both forms of anxious attachment [16,57], are associated with less satisfaction with the couple’s relationship, less sexual satisfaction, and the use of maladaptive coping mechanisms in the face of couple crises. An important pattern of anxious attachment is hyperactive attachment systems. In this case, a sensitive and hypervigilance position is activated in the relationship, and there might be appears some signs of obsessive love [26,58]. Fearful attachment is associated with a greater likelihood of separation or divorce, while preoccupied attachment is associated with greater difficulty in ending the relationship even in conditions of malaise [59,60,61,62,63].

Furthermore, our results also highlighted an indirect path in the relationship between fearful and preoccupied adult attachment styles and love addiction, through the mediation effect of self-esteem, confirming all the hypotheses (**H_1_** to **H_3_**). More specifically, fearful and preoccupied attachment have been negatively associated with self-esteem (**H_1_**), as both of them are indeed characterized by a negative view of self, coupled with a negative and positive view of the other [16], which can result in lower levels of self-esteem. Consistently, the scientific literature shows that both preoccupied and fearful attachment styles are associated with lower levels of self-esteem than secure and dismissing attachment styles [33]; an anxious type of attachment would seem to be more associated, than the secure attachment style, with an unclear self-concept and more unstable self- esteem [30,31,64]. In turn, results shown a negative association between self-esteem and love addiction (**H_2_**), as low self-esteem can manifest itself in dysfunctional relational modes, also being a contributing factor to the formation of interpersonal dependence [36]. This is in line with previous studies that highlight associations between low self-esteem and low couple satisfaction, as individuals with low self-esteem believe that the partner sees them as they see themselves; therefore, to avoid disappointment, they tend to distance themselves. This leads to a reduction in couple satisfaction for both partners [65,66]. Moreover, this is in line with previous associations that highlight how low self-esteem is a risk factor for addictive behaviours, such as Internet, social media, and exercise addiction [34,35,67,68,69,70] and, more generally, is associated with lower levels of well-being, with more negative effects, fewer positive effects, and greater stress severity [71]. Therefore, the results of this study highlight that individuals with higher levels of fearful or preoccupied attachment, characterized by a negative self-view [16], tend to show lower levels of self-esteem which can result in dysfunctional relational modalities characterized by an excessive dependence on the partner [36] (**H_3_**). 

Finally, the role of gender and age as potential confounders was controlled in testing the model. Focusing on gender, being female was associated with low levels of self-esteem and high levels of love addiction. This is in line with previous research showing how women tend to have lower self-esteem than men, especially for the domains of physical appearance, athleticism, personal self, and self-satisfaction [38,39,72]. Furthermore, this is consistent with previous research which highlighted the greater tendency in women than men to be addicted to love and relationships [4]. Concerning age, this factor did not show a direct effect on love addiction, while being younger was associated with lower levels of self-esteem. This could be read in relation to studies which highlight that self-esteem in young adults tends to be lower than in adulthood; in fact, instability and contingency of self-esteem decrease from adolescence to old age, whereas levels of self-esteem increase. Findings suggest that people’s self-esteem tends to become better adjusted (i.e., more stable, less contingent, and higher) across their life course [39,73]. In addition, young people seem to have less self-efficacy and less belief in their own potential for change, and generally report a lower level of well-being than older people [74,75,76].

The present study also has some limitations that should be highlighted. First, the study design was cross-sectional, and this requires caution in the interpretation of the causal link between the variables, as well as the directionality of these relationships. Therefore, although a solid basis of the scientific literature was used for the implementation of the model, it is also plausible that love addiction, in turn, influences self-esteem again, resulting in a vicious circle. Given this possibility, the replication of these results in longitudinal studies is needed in future research. Furthermore, the gender imbalance in the sample should be considered when interpreting the results. Although this issue was limited by controlling the effect of gender when testing the model, the use of more balanced samples could be an important challenge for future research. Finally, the use of self-report measures requires participants to be self-observing and willing to express aspects of themselves, exposing them to a variety of biases, e.g., the desirability bias. Although previous research on the scale employed here shows their good psychometric properties, theoretical foundation, and their excellent ability to discriminate the constructs, the use of a multi-method approach (e.g., integrating the assessment with interviews) could enrich the study and make future research more stable.

## 5. Conclusions

Given the negative and pervasive impact of love addiction in individuals’ lives [3], this research has focused on the variables that may be associated with this condition, providing information that can be useful to support and guide clinical practice. First, the results highlight the significant influence of fearful and preoccupied adult attachment styles. This data supports the possibility of directing the therapeutic work with subjects with love addiction on these factors, especially in the light of the broad line of research that highlights the effectiveness of clinical treatments in promoting changes in attachment patterns [77,78,79]. Furthermore, findings also showed the influence of self-esteem, which played a full mediator role in the model and presented negative associations with love addiction. This further corroborates the role of self-esteem in promoting mental health [80] and suggests the importance of focusing on this variable in both preventive and clinical practice.

## Figures and Tables

**Table 1:** Demographic characteristics of the sample (*N* = 300).

Characteristics		*M ± SD*	*N*	*%*
Age		37.83 ± 12.937		
Sex				
	*Males*		60	20
	*Females*		240	80
Marital Status				
	*Cohabiting*		175	58
	*Married*		125	42
Education				
	*Middle school diploma*		21	7
	*High school diploma*		114	38
	*University degree*		76	25
	*Master’s degree*		60	20
	*Post-lauream specialization*		29	10
Occupation				
	*Student*		32	11
	*Working student*		34	11
	*Artisan*		6	2
	*Homemaker*		8	3
	*Trader*		8	3
	*Employee*		138	46
	*Manager*		10	3
	*Entrepreneur*		15	5
	*Freelance*		22	7
	*Retired*		9	3
	*Unemployed*		18	6

***Note:****N* = Number; *M* = Mean; *SD* = Standard Deviation.

**Table 2:** Correlation matrix.

	1	2	3	4	5	6	7
1. Love addiction	1						
2. Secure attachment	−0.051	1					
3. Preoccupied attachment	0.203 **	−0.200 **	1				
4. Fearful attachment	0.256 **	−0.073	0.253 **	1			
5. Dismissing attachment	−0.105	−0.200 **	0.014	−0.061	1		
6. Self-esteem	−0.364 **	0.266 **	−0.319 **	−0.413 **	0.118 *	1	
7. Age	−0.163 **	−0.026	−0.224 **	−0.141 *	0.012	0.277 **	1
8. Gender	−0.040	0.065	0.057	0.041	−0.219 **	−0.165 **	−0.169 **

***Note***: bold values indicate significant *p*-values; ** correlation is significant at the 0.01 level (2-tailed); * correlation is significant at the 0.05 level (two-tailed). Gender—males are coded as “0”, and females are coded as “1”.

**Table 3:** Coefficients of the structural equation mediation model.

	Estimate	SE	*p*	Boot LLCI	Boot ULCI
*Total effects*					
Preoccupied attachment → love addiction	0.129	0.056	<0.01	0.050	0.239
Fearful attachment → love addiction	0.212	0.064	<0.01	0.111	0.312
*Direct effects*					
Preoccupied attachment → love addiction	0.072	0.056	0.182	−0.013	0.172
Fearful attachment → love addiction	0.112	0.066	0.083	−0.008	0.225
*Indirect effects*					
Preoccupied attachment → self-esteem → love addiction	0.056	0.023	<0.01	0.023	0.099
Fearful attachment → self-esteem → love addiction	0.100	0.030	<0.01	0.061	0.172

## Data Availability

The data presented in this study are available on request from the corresponding author. The data are not publicly available due to privacy reasons.
